# Sex and Gender Bias in Covid-19 Clinical Case Reports

**DOI:** 10.3389/fgwh.2021.774033

**Published:** 2021-11-22

**Authors:** Aysha E. Salter-Volz, Abigail Oyasu, Chen Yeh, Lutfiyya N. Muhammad, Nicole C. Woitowich

**Affiliations:** ^1^Department of Medical Social Sciences, Feinberg School of Medicine, Northwestern University, Chicago, IL, United States; ^2^University of Illinois at Urbana-Champaign, Champaign, IL, United States; ^3^Division of Biostatistics, Department of Preventive Medicine, Feinberg School of Medicine, Northwestern University, Chicago, IL, United States

**Keywords:** COVID-19, sex bias, gender bias, case reports (publication type), bibliometrics

## Abstract

Clinical case reports circulate relevant information regarding disease presentation and describe treatment protocols, particularly for novel conditions. In the early months of the Covid-19 pandemic, case reports provided key insights into the pathophysiology and sequelae associated with Covid-19 infection and described treatment mechanisms and outcomes. However, case reports are often subject to selection bias due to their singular nature. To better understand how selection biases may have influenced Covid-19-releated case reports, we conducted a bibliometric analysis of Covid-19-releated case reports published in high impact journals from January 1 to June 1, 2020. Case reports were coded for patient sex, country of institutional affiliation, physiological system, and first and last author gender. Of 494 total case reports, 45% (*n* = 221) of patients were male, 30% (*n* = 146) were female, and 25% (*n* = 124) included both sexes. Ratios of male-only to female-only case reports varied by physiological system. The majority of case reports had male first (61%, *n* = 302) and last (70%, *n* = 340) authors. Case reports with male last authors were more likely to describe male patients [*X*^2^ (2, *n* = 465) = 6.6, *p* = 0.037], while case reports with female last authors were more likely to include patients of both sexes [OR = 1.918 (95% CI = 1.163–3.16)]. Despite a limited sample size, these data reflect emerging research on sex-differences in the physiological presentation and impact of Covid-19 and parallel large-scale trends in authorship patterns. Ultimately, this work highlights potential biases in the dissemination of clinical information via case reports and underscores the inextricable influences of sex and gender biases within biomedicine.

## Introduction

Longstanding sex and gender biases impact many facets of the biomedical research enterprise including research practices (1, 2), clinical care (3, 4), and workforce development (5–7). The persistent overrepresentation of males as research subjects, scientists, and physicians has informed our understanding of health and disease, oftentimes to the detriment of women, transgender, and gender non-binary, or non-conforming individuals.

Clinical case reports serve as an important educational tool to disseminate pertinent information regarding disease or disorder presentation, diagnosis, treatment, and prognosis (8). During the initial months of the Covid-19 pandemic, case reports provided key insights into Covid-19 pathophysiology, sequelae, and treatment and in certain circumstances, served as primary evidence for clinical decision-making.

Case report subjects are often selected semi-retrospectively for their novelty or educational benefit. As a result, singular case reports are inherently prone to selection bias. In contrast to clinical research studies which have predefined study populations and stringent inclusion or exclusion criteria, the decision to select a case report subject may lie solely with a member of the patient's care team. However, it is reasonable to expect that if case studies were compiled for a particular disease or disorder, they would closely mirror the respective patient population. In 2017, Allotey and colleagues (9) identified a significant male bias in case reports published in high-impact medical journals, which suggests that inherent biases may play a larger role than anticipated in case report selection and publication. We hypothesized that female patients may be underrepresented in Covid-19 research and clinical care due to sex differences in Covid-19 disease or due to gender biases. To determine whether Covid-19-related case reports were, in fact, subject to sex or gender biases, we characterized 494 Covid-19-related case reports published between January 1, 2020, and July 1, 2020, from 103 journals by patient sex, physiological system, country of institutional affiliation and first and last author gender.

## Methods

**Figure 1:**
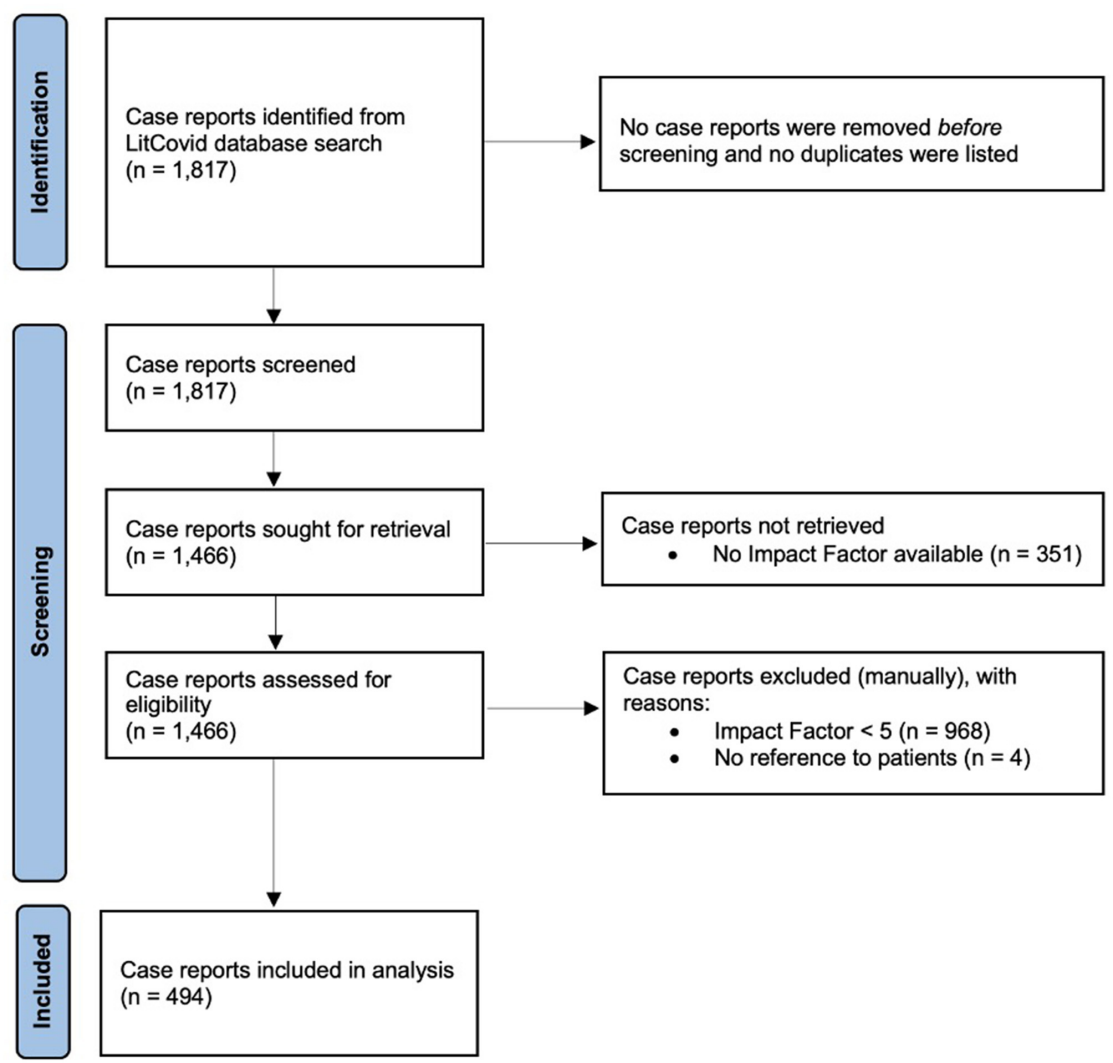
Study flow diagram of the identification and screening of eligible Covid-19-related case reports retrieved from the PubMed LitCovid database.

Citation data for 1,817 articles classified as case reports were downloaded on July 1, 2020, from LitCovid, a categorical database of Covid-19 literature from PubMed (10, 11). The LitCovid database identifies relevant articles using the National Center for Biotechnology Information's E-Utilities tool which is then further refined and categorized by machine learning and manual creation (11). Case reports were further refined by additional inclusion and exclusion criteria (Figure 1). Journal impact factors [(IF), *2019 Journal Citation Reports Science Edition*, Clarivate Analytics] were available for 1,466 (81%) case reports, and only those with an IF of 5 or above (*n* = 498, 27%) were considered medium-to-high visibility and selected for inclusion in the study and further review. Four articles were excluded because they did not reference patients, resulting in a final sample of 494 articles. Two of the authors (ASV, AO) manually and independently screened and coded case reports for patient sex, physiological system, author first names, and country of institutional affiliation. Patient sex was determined by the use of descriptive terms such as male/female, man/woman, or inferred by the use of he/she pronouns. Only one article (0.2%) included transgender patients and did not report biological sex or gender identity. The country of institutional affiliation was determined by the institutional location of the corresponding author if the article was authored by a multinational cohort. These data were cross-checked, and the coding agreement was almost perfect for a representative subset of 55 articles (Cohen's kappa = 0.97, *p* < 0.001). The first and last author's gender were inferred using the name-to-gender assignment algorithm Gender API (https://gender-api.com/). Gender API was selected due to its low rate of inaccuracies (7.9%) or non-classifications (3%) (12). Articles authored by an unspecified group or without full first names listed were coded as unknown.

Chi-Square tests and multinomial logistic regression models were used to examine the association between author gender and patient sex. Chi-Square tests were also used to compare patient sex by physiological system and country of institutional affiliation. Results from the multinomial logistic regression models are summarized by odds ratios (OR) and 95% confidence intervals (CI). *P*-values < 0.05 were considered significant.

Descriptions of patient sex or author gender follow American Psychological Association reporting standards where male/female terminology is used as descriptive adjectives when appropriate or when specifically referring to biological sex. The terms men and women are commonly used as nouns to describe groups of people.

## Results

**Figure 2:**
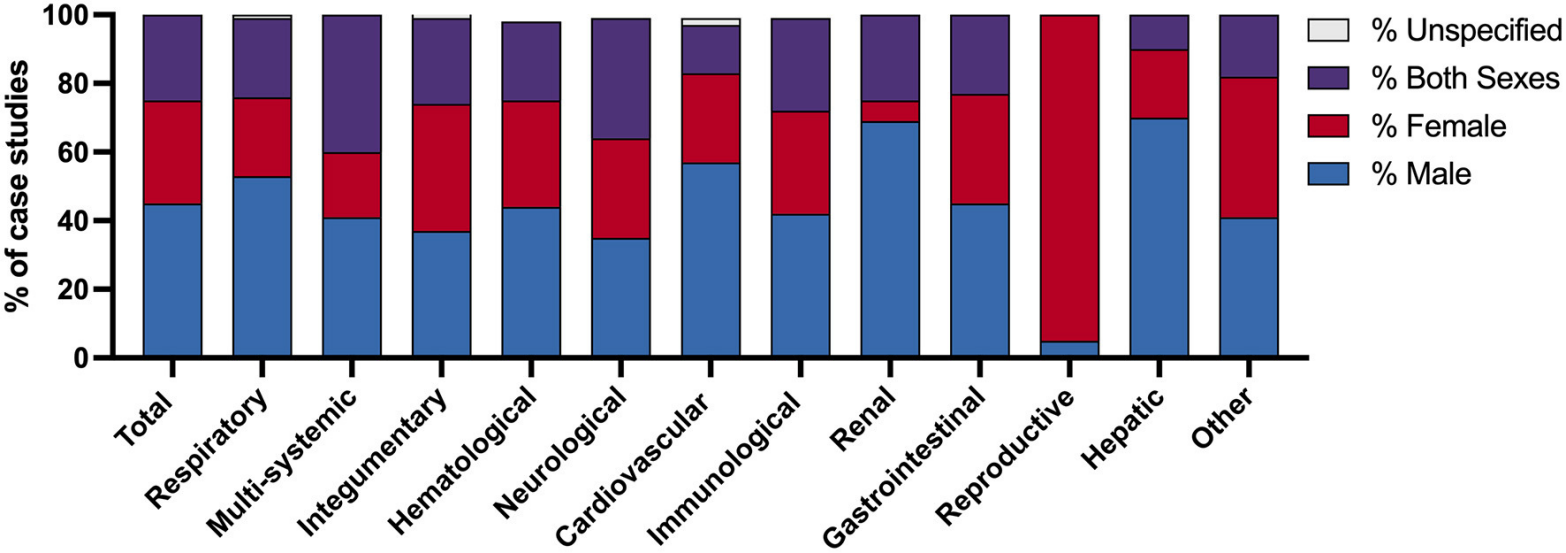
Comparison of Covid-19 case studies by patient sex and physiologic body system. The percentage of Covid-19 case studies which describe patient sex as male, female, both sexes, and unspecified. Data are presented by the category of case study, coded by physiologic body system, as well as the sum of all case studies evaluated.

Of the 494 case reports analyzed, the majority were related to respiratory, multi-systemic, dermatologic, hematologic, or neurologic systems (Table 1). Of the patients described in the 494 case reports, forty-five % (*n* = 221) were male and 30% (*n* = 146) were female (Figure 2). Patients of both sexes were included in 25% (*n* = 124) of case reports and 0.6% (*n* = 3) failed to report patient sex (Figure 1). The ratio of articles reporting on male-only vs. female-only patients was highest in renal (11:1), hepatic (3.5:1), respiratory (2.3:1), multi-systemic (2.2:1), and cardiovascular (2.2:1) systems. Reproductive reports were almost exclusively female (95%, *n* = 20).

**Table 1:** Case study characteristics and article metadata.

	**Total**		**Male only**		**Female only**		**Both sexes**		**Chi-square test *p*-value**
	***N***	**(%)**	***N***	**(%)**	***N***	**(%)**	***N***	**(%)**	
	494	(100)	221	(100)	146	(100)	124	(100)	
**Body system**									<0.001
Respiratory^*^	74	(15)	39	(18)	17	(12)	17	(14)	
Multi-system	70	(14)	29	(13)	13	(9)	28	(23)	
Integumentary^*^	61	(12)	22	(10)	22	(15)	16	(12)	
Hematological	47	(10)	21	(10)	15	(10)	11	(9)	
Neurological	48	(10)	17	(8)	14	(10)	17	(14)	
Cardiovascular^*^	42	(9)	24	(11)	11	(7)	6	(5)	
Immunological	33	(7)	14	(6)	10	(7)	9	(7)	
Renal	32	(6)	23	(10)	2	(1)	8	(6)	
Gastrointestinal	22	(4)	10	(5)	7	(5)	5	(4)	
Reproductive	21	(4)	1	(0)	20	(14)	0	(0)	
Hepatic	10	(2)	7	(3)	2	(1)	1	(1)	
Other	34	(7)	14	(6)	14	(10)	6	(5)	
**Country of institutional affiliation**									0.294
USA	97	(20)	37	(17)	32	(22)	28	(23)	
China	89	(18)	38	(17)	20	(14)	31	(25)	
Italy	65	(13)	32	(14)	21	(14)	12	(10)	
France	60	(12)	25	(11)	17	(11)	15	(12)	
Spain	34	(7)	14	(6)	12	(8)	8	(6)	
Other	149	(30)	75	(34)	45	(31)	30	(24)	
**First author gender**									0.639
Male	302	(61)	137	(62)	92	(63)	72	(58)	
Female	182	(39)	77	(35)	53	(36)	50	(40)	
Unknown	10	(2)	7	(3)	1	(1)	2	(2)	
**Last author gender**									0.037
Male	340	(69)	155	(70)	104	(71)	79	(58)	
Female	128	(26)	44	(20)	40	(27)	43	(40)	
Unknown	26	(5)	22	(10)	2	(1)	2	(2)	
**Author dyads**	457	(100)	194	(100)	143	(100)	120	(100)	0.135
Male first / male last	214	(43)	96	(50)	71	(33)	47	(39)	
Male first / female last	72	(15)	28	(14)	19	(26)	25	(21)	
Female first / male last	116	(24)	54	(28)	32	(28)	30	(25)	
Female first / female last	55	(11)	16	(8)	21	(38)	18	(15)	

* *Sex unspecified for one article in each of the following categories: respiratory, integumentary, and cardiovascular*.

Case reports were primarily authored by groups with institutional affiliations in the United States (20%, *n* = 97), China (18%, *n* = 89), Italy (13%, *n* = 65), France (12%, *n* = 60), and Spain (7%, *n* = 34). The majority of case reports had male first (61%, *n* = 302) and last (70%, *n* = 340) authors, with 43% (*n* = 214) of all reports having male first and last author dyads. The last author's gender is associated with the sex of the case report patient (Table 1). Case reports with male last authors are more likely to include male-only patients (*p* = 0.037) compared to female last authors. Female last authors are more likely to include patients of both sexes [OR = 1.918 (95% CI = 1.163–3.16)] in unadjusted and adjusted models [OR = 1.774 (95% CI = 1.055–2.984)] which control for impact factor, country, and physiological system.

## Discussion

While male bias in case reports has been previously reported (9), this is the first study to examine this in Covid-19-related case studies. The overrepresentation of male patients in Covid-19 case reports may be reflective of sex differences in disease prevalence, severity, and immune response (13, 14). Likewise, sex and gender differences in the presence of contributing comorbidities may also influence Covid-19 disease severity and treatment outcomes (15). The high ratio of male-to-female case reports in the renal category parallels clinical data which suggests that male sex risk factor for Covid-19-related acute kidney injury (15, 16). In comparison, the high female-to-male ratio observed in the reproductive category can be attributed to pregnancy-related case reports. Overall, the differences in patient sex ratios across physiological categories may provide insight into Covid-19 disease mechanisms. Yet, it is important to note that these data fail to fully capture the sociocultural influences on Covid-19 testing, case identification, and access to care which may differ based on gender, race, ethnicity, socioeconomic status, and geographic location as case reports typically originate from a hospital-based setting.

Gender disparities in authorship are common within the biomedical sciences (17, 18) and have been documented for case reports (19). In a large-scale bibliometric analysis of over 20,000 case reports, Hsiehchen and colleagues (19) found that 36% of first authors and 25% of last authors are women. The data presented here are similar with female authors comprising 39% and 26% of first and last authors, respectively. Of interest, is the unique influence of the Covid-19 pandemic on gender authorship patterns. Early in the pandemic, several groups reported that women were publishing less to biomedical preprint servers compared to the same period in 2019 (20, 21). Meanwhile, others found that women were underrepresented as first authors on Covid-19-related research studies (22, 23). The case reports analyzed here were authored during the first 6 months of the pandemic yet reflect pre-pandemic authorship trends. This suggests that authorship trends should not solely be used as a metric for assessing the impact of Covid-19 on research productivity and more long-term, holistic evaluations of the biomedical enterprise are warranted. In depth analyses which evaluate other metrics of productivity such as grant submission and award patterns and hiring, retention, and promotion rates, at discipline- or specialty-specific levels and the availability and/or accessibility of institutional support structures would provide added insight into the impact of Covid-19 on the biomedical workforce.

Lastly, emerging evidence suggests that author gender may also influence how data are analyzed and presented (24, 25). Prior work by Sugimoto and colleagues found that women are more likely to report and analyze data by sex (25). Here, we find that female authors are more likely to include patients of both sexes within case reports. These data suggest that female authors may be more likely to find inherent value in including clinical data derived from both sexes in case reports. Alternatively, they may be more keenly aware of, and actively seek to address sex- and gender biases in biomedicine through inclusivity. On the contrary, case reports with male last authors were more likely to include male-only patients. As last authorship generally confers seniority and intellectual leadership, these data suggest that sex or gender biases held by the senior author, whether implicit or explicit, may influence the selection of case report patients and reporting outcomes. The male-bias observed in Covid-19 case reports may be reflective of the patient population, as men who are diagnosed with Covid-19 are more likely to require hospitalization and critical care (26), however it does not fully explain the authorial differences in case report selection. The fact that female authors are more likely to include patients of both sexes is of interest and warrants further examination particularly in disease areas which are predominantly sex-specific.

This study is not without limitations. First, the sample size of case reports analyzed was limited due to stringent inclusion criteria related to journal impact factor and date of publication; although it is important to note that this sample size remains a significant and representative subset of the original sample of case reports. Journal impact factors of 5 and above were selected to represent case reports likely to be of medium-to-high visibility within the biomedical community. However, we recognize that journal impact factors are variable across biomedical disciplines and medical specialties and serve only as one metric to assess the quality, impact, and visibility of an article. As a result, case reports published in journals related to obstetrics and gynecology and reproductive health were likely omitted due to traditionally lower impact factors. The inclusion of case reports from women's health-related journals may have made the data appear more balanced and less suggestive of a sex-bias. Yet, by excluding these articles the data more broadly reflects sex and gender biases that exist outside of sex-specific fields of medicine, although we recognize that obstetric, gynecologic, and reproductive care is provided to those who identify across the gender spectrum.

In addition, these data were collected from the first 6 months of the Covid-19 pandemic, during which time the diagnosis, treatment, and understanding of the disease were rapidly evolving. We therefore cannot quantify the potential biases associated with clinical care that occurred later in the pandemic. The *in silico* tools to assign author gender also present another limitation as these are currently limited to gender binary options (male, female, or unknown) and therefore exclude or misrepresent the identity of those who are gender non-binary, non-conforming, two-spirit, or third gender. Moreover, some case reports did not explicitly define patient's sex or gender. For coding purposes, patient sex for these was inferred through the use of terms such as man/woman, male/female, or descriptive he/she pronouns, and there may be instances where patient sex and gender identity do not correspond. Often the terms “sex” and “gender” were used interchangeably within case reports, making it difficult to separate patient's biological sex from their gender identity. The distinctions of both biological sex and gender should be noted in case reports, as gender is a contributing social determinant of health.

## Conclusion

The associations between author gender and patient sex suggests that sex or gender biases are contributing factors which impact patient reporting. The coordinated efforts of clinicians, reviewers, editors, and publishers are required to ensure a balanced representation of the relevant patient population. Gender has been widely recognized as a social determinant of health and as such gender biases can contribute to gender-based health disparities. Diversification of the biomedical workforce appears to be critical, but rate-limiting factor, in reducing sex- and gender biases that permeate biomedicine. As more gender-diverse perspectives are included in the selection, writing, reviewing, and publishing of case reports, their subsequent quality, and educational value are likely to improve. Acknowledging and actively addressing biases may further a better understanding of the influences of sex and gender on health and disease, ultimately minimizing health disparities.

## Data Availability Statement

The raw data supporting the conclusions of this article will be made available by the authors, without undue reservation.

## Author Contributions

NW: full access to all the data in the study and takes responsibility for the integrity of the data and the accuracy of the data analysis, obtained funding, administrative, technical, or material support, supervision, concept, and design. AS-V, AO, and NW: drafting of the manuscript. CY, LM, and NW: statistical analysis. All authors: acquisition, analysis, or interpretation of data and critical revision of the manuscript for important intellectual content.

## Funding

This work was supported by a Women's Health Access Matters Grant to NW. The funders had no role in the design and conduct of the study; collection, management, analysis, and interpretation of the data; preparation, review, or approval of the manuscript; and decision to submit the manuscript for publication.

## Conflict of Interest

The authors declare that the research was conducted in the absence of any commercial or financial relationships that could be construed as a potential conflict of interest.

## Publisher's Note

All claims expressed in this article are solely those of the authors and do not necessarily represent those of their affiliated organizations, or those of the publisher, the editors and the reviewers. Any product that may be evaluated in this article, or claim that may be made by its manufacturer, is not guaranteed or endorsed by the publisher.
